# Soluble Polyimides Bearing (*cis, trans*)-Hydrogenated Bisphenol A and (*trans, trans*)-Hydrogenated Bisphenol A Moieties: Synthesis, Properties and the Conformational Effect

**DOI:** 10.3390/polym11050854

**Published:** 2019-05-10

**Authors:** Zhiming Mi, Shuai Wang, Ziwen Hou, Zhixiao Liu, Sizhuo Jin, Xiaowen Wang, Daming Wang, Xiaogang Zhao, Yumin Zhang, Hongwei Zhou, Chunhai Chen

**Affiliations:** 1 Key Laboratory of High Performance Plastics (Jilin University), Ministry of Education. National & Local Joint Engineering Laboratory for Synthesis Technology of High Performance Polymer. College of Chemistry, Jilin University, Changchun 130012, China; mizm16@mails.jlu.edu.cn (Z.M.); wangshuai1215@mails.jlu.edu.cn (S.W.); houzw18@mails.jlu.edu.cn (Z.H.); lzx16@mails.jlu.edu.cn (Z.L.); g877320403@gmail.com (S.J.); wxw17@mails.jlu.edu.cn (X.W.); wangdaming@jlu.edu.cn (D.W.); xiaogang@jlu.edu.cn (X.Z.); cch@jlu.edu.cn (C.C.); 2 College of Chemistry, Jilin University, Changchun 130012, China; zhang_ym@jlu.edu.cn

**Keywords:** HBPA isomers, transparent, soluble, polyimide

## Abstract

In this work, hydrogenated bisphenol A (HBPA) based dinitro mixed isomers (1a′ and 1a) were synthesized and separated via vacuum distillation under the monitor of DSC and ^1^H NMR. Corresponding diamines (2a′ and 2a) were separately polycondensed with five commercial dianhydrides via a two-step thermal imidization to obtain PI-(1′-5′) and PI-(1-5). All the polyimides could afford flexible, tough, and transparent films, and most of them were readily soluble not only in common polar solvents like DMAc, but also in low boiling point solvents such as chloroform. ^1^H NMR spectra of the polyimides demonstrated that HBPA moiety showed no conformation changes during the preparation of polymers. For a given dianhydride, PI-(1-5) exhibited better thermal stability than that of PI-(1′-5′), this can be attributed that the *equatorial, equatorial* C–O in PI-(1-5) promoted denser and more regular molecular chain stacking, as can be evidenced by the WAXD and geometric optimization results. Additionally, when the dianhydride was ODPA, BPADA or 6FDA, no apparent difference was found in either the transmittance or solubility between two series of polyimides, which could be attributed that twisted and flexible ether linkages, as well as bulky substituents, led to the “already weakened” inter- and intramolecular CT interaction and cohesive force. However, when it came to rigid and stiff dianhydride, e.g., BPDA, PI-3′ took an obvious advantage over PI-3 in transmittance and solubility, which was possibly owed to the larger molecular chain d-spacing imparted by *equatorial, axial* C–O. An overall investigation of PI-(1′-5′) and PI-(1-5) on aspects of thermal, mechanical, morphological, soluble and optical performance values was carried out, and the conformation effects of HBPA isomers on the properties of two series of polyimides were discussed in detail.

## 1. Introduction

Aromatic polyimides possess outstanding properties such as mechanical toughness, high-temperature resistance, dimensional stability, low dielectric value, radiation resistance, selectivity to gases, etc. [1,2,3,4]. Therefore, they are indispensable participants in various fields including the aerospace, microelectronics, nuclear industry and gas separation membrane fields. Nevertheless, disadvantages of aromatic polyimides including the pale yellow to brown color as well as the difficulty in the processability should be taken into consideration before they can be made suitable for optoelectronic applications [5].

Classically, intra- and intermolecular charge transfer (CT) interactions between the electron-donating diamine and electron-accepting aromatic dianhydride are considered the main reason for the coloration of aromatic polyimides, and the stiff and ordered structure of the molecular chains led to the insolubility or infusibility of aromatic polyimides, which deteriorate their processing and applications [6,7,8,9]. In this regard, extensive reports [10,11,12,13] have focused on the structure modification of polyimides, e.g., the introduction of a fluorinated structure, cycloaliphatic segments, bulky pendant substituents, flexible and unsymmetrical linkages and electron withdrawing chlorine atoms in the backbone of polymers, aiming at reducing several types of chain-chain interactions such as CT complexes, electronic polarization interactions and chain packing (e.g., crystallinity) to obtain colorless and soluble polyimides. Of significant interest and widely studied approaches is the integration of alicyclic fragments in the backbone of polyimides [7,14,15,16,17]. Owing to the non-conjugated, non-planar, rigid characterizations of the alicyclic structure, the introduction of these fragments in the backbone of polyimides can simultaneously impede the formation of intra- and intermolecular CT complexes and weaken the cohesive force between molecular chains to improve the transmittance and solubility of the polyimide films without compromising the thermal and mechanical properties.

Hydrogenated bisphenol A (HBPA) is promising and reveals alicyclic candidates to offer diamine or dianhydride monomer. Due to the different spatial conformations of hydroxyls, HBPA contains three isomers, (*trans, trans*)-HBPA, (*cis, trans*)-HBPA and (*cis, cis*)-HBPA. Seen from their steric conformations presented in Figure S1, HBPA moiety owns a distorted and asymmetric structure, and mainly consists of two “chair shaped” cyclohexane rings which are linked by isopropyl. Moreover, Figure S2 presents the GC-MS of HBPA isomers, and the content distribution is (*cis, cis*)-HBPA, 8%; (*cis, trans*)-HBPA, 45%; (*trans, trans*)-HBPA, 47%. As can be deduced, (*trans, trans*)-HBPA is the most stable conformation because both hydroxyls are located at the *equatorial* bond of the cyclohexane, while (*cis, cis*)-HBPA is less stable due to the repulsive force between the *axial* hydroxyls and *axial* hydrogens in cyclohexane. Over the years, there have been numerous reports on the incorporation of cyclohexane into the backbone of polyimides to obtain highly transparent, readily soluble polyimides. S. Ando et al. [18] utilized a-BPDA/CHDA to developed colorless and processable semialiphatic polyimide films; Hasegawa et al. [19] synthesized 1S,2S,4R,5R-cyclohexanetetracarboxylic dianhydride (H′PMDA), and H′PMDA based polyimides were approximately colorless with a transmittance of up to 90.3% at 400 nm; Fang et al. [20] developed *cis*-1,2,3,4-cyclohexanetetracarboxylic dianhydride (*cis*-1,2,3,4-CHDA), and polyimides bearing *cis*-1,2,3,4-CHDA units presented good thermal, mechanical, and soluble properties, and the cut-off wavelength was around 270 nm. These results suggested that the cyclohexane moiety in the backbone of polyimides promoted the ideal properties of the polymer. However, rare reports of HBPA based polyimides were found, which can be ascribed two aspects: firstly, commercialized HBPA is different in composition due to the different hydrogenation conditions (catalyst type, temperature, etc.) of BPA, so the molar ratio of the three isomers in HBPA is different. Therefore, the polyimide property containing the HBPA structure is not fixed, which owns no research value. The second aspect was probably ascribed to the difficulty in separating HBPA isomers. In our previous work [21], (*trans, trans*)-HBPA was separated from HBPA isomers via recrystallization, and (*trans, trans*)-HBPA based diamine and dianhydride were successfully synthesized. HBPA based polyimides were colorless with a transparency as high as 86% at 450 nm, and soluble in low boiling point solvents such as dichloromethane. However, the separation of (*cis, trans*)-HBPA and (*trans, trans*)-HBPA based monomers, as well as their conformation effects on the overall performance of polyimides still remain a challenge.

In this paper, HBPA based dinitro mixed isomers (1a′ and 1a) were synthesized, (*cis, trans*)-bis(4-nitrophenoxy)HBPA (1a′) and (*trans, trans*)-bis(4-nitrophenoxy)HBPA (1a) were separated via vacuum distillation under the monitor of DSC and ^1^H NMR. After reduction, two diamines, (*cis, trans*)-bis(4-aminophenoxy)HBPA (2a′) and (*trans, trans*)-bis(4-aminophenoxy)HBPA (2a) were polycondensed with five kinds of commercial dianhydrides to obtain PI-(1′-5′) and PI-(1-5), respectively. The chemical structures of monomers and polyimides were characterized by HRLC-MS, FTIR, ^1^HNMR, ^13^C NMR and 2D NMR. The main purpose of introducing the HBPA moiety into the polyimide backbone includes two aspects: firstly, alicyclic HBPA was expected to weaken the molecular chain cohesion force and impede CT interactions, hence, resulting in the improved processability and alleviated coloration of polyimides. Secondly, to clarify the conformation effects of HBPA isomers on the properties of two series of polyimides. To the best of our knowledge, this is the first time that the conformation effects of HBPA isomers are explored in a set of different polyimides with varying dianhydride architecture.

## 2. Experimental

### 2.1. Materials

HBPA (a mixture of isomers, (*trans, trans*)-HBPA: 47%; (*cis, trans*)-HBPA: 45%; (*cis, cis*)-HBPA: 8%;) was purchased from Kesheng Chemical Co., Ltd., Rongcheng, China. The compound 4-Fluoronitrobenzene (98%) and 60 wt % sodium hydride (NaH) were bought from Aldrich (Shanghai, China). Methanol, dioxane, 10 wt % palladium on charcoal (Pd/C) and 80 wt % hydrazine hydrate (N_2_H_4_·H_2_O) were obtained from Sinopharm Chemical Reagent Co., Ltd., Shanghai, China. Commercial dianhydrides including 3,3′,4,4′-oxydiphthalic anhydride (ODPA), 3,3′,4,4′-biphenyltetracarboxylic dianhydride (BPDA), 4,4′-(hexafluoroisopropylidene)-diphthalic anhydride (6FDA), 4,4′-(4,4′-isopropylidenediphenoxy)bis(phthalic anhydride) (BPADA), were provided by the Tokyo Chemical Industry (TCI) Co., Ltd., Tokyo, Japan. The compound 3,3′,4,4′-Benzophenonetetracarboxylic dianhydride (BTDA) was supplied by Evonik Jayhawk Fine Chemicals, Galena, KS, USA. *N, N*-Dimethylformamide (DMF) and *N, N*-dimethylacetamide (DMAc) were purified by vacuum distillation over CaH_2_ and stored over 4 Å molecular sieves prior to use. All the commercially available reagents and solvents were directly used unless specified.

### 2.2. Characterization

Fourier transform infrared spectroscopy (FTIR) were determined by a Bruker Vector 22 spectrometer at a resolution of 4 cm^−1^ in the range of 4000–500 cm^−1^. The ^1^H NMR (nuclear magnetic resonance, 300 MHz) and ^13^C NMR (75 MHz) spectra were performed using a BRUKER-300 spectrometer (Bruker, Hamburg, Germany) with tetramethylsilane (TMS) as the internal standard and deuterated dimethylsulfoxide (DMSO-d_6_) as the solvent. Two-dimensional NMR spectra including correlation spectra (COSY) and heteronuclear single quantum correlation (HSQC) were recorded on a BRUKER-AVANCEIII500 (500 MHz) (Bruker, Hamburg, Germany) at 300 K with technical specifications of ^1^H S/N ≥ 760:1 (0.1% ethylbenzene in CDCl_3_) ^13^C S/N>500:1 (ASTM). The NMR data were processed using the Topspin 3.2 software and all chemical shifts were given in ppm and coupling constants in Hz. High-resolution liquid chromatography-mass spectrometry (HRLC-MS) data were obtained using Agilent 1290-micrOTOF-QII (Agilent, Santa Clara, CA, USA). Inherent viscosities (η_inh_) of PAA were estimated using an Ubbelohde viscometer at 25 °C in DMAc at a concentration of 0.5 g dL^−1^. Weight-average molecular weights (M_w_) and number-average molecular weights (M_n_) were measured by gel permeation chromatography (GPC) on the basis of polystyrene calibration on a PL GPC 220 instrument (Agilent, Santa Clara, CA, USA) equipped with a refractive index detector using DMF as an eluent at a flow rate of 1.0 mL min^−1^. The used GPC column was 3 × PLgel 10 µm MIXED-B, 300 × 7.5 mm. Differential scanning calorimetric (DSC) analysis was conducted on a TA instrument DSC Q100 (TA, New Castle, DE, USA) at a scanning rate of 10 °C min^−1^ under nitrogen with a gas flow of 50 mL min^−1^. For the test of the melting point of the monomer, the first heating scans were taken as the final data; for the test of glass transition temperature (T_g_) of the polyimide, the second heating scans were taken as the final data. Dynamic mechanical analysis (DMA) was carried out on the polyimide film specimens (length, 10 mm; width, 6 mm; thickness, 45 µm) using a TA instrument DMA Q800 (TA, New Castle, DE, USA) with a load frequency of 1 Hz and a heating rate of 5 °C min^−1^ from room temperature to 330 °C in air. Glass transition temperature (T_g_) was determined as the peak temperature of the tan δ curve. Thermo gravimetric analysis (TGA) was carried out on TA 2050 under air or nitrogen atmosphere with a heating rate of 10 °C min^−1^. Wide-angle X-ray diffractometer (Empyrean, PANalytical B.V.) equipped with a PIXcel^3D^ all-around matrix detector was employed to evaluate morphological of polyimide films and the diffraction patterns were collected in the reflection mode over 2θ ranging from 5° to 50°. Mechanical properties were performed on a Shimadzu AG–I universal testing apparatus (Shimadzu, Kyoto, Japan) with a crosshead speed of 5 mm min^−1^, tensile modulus (T_M_), tensile strength (T_S_), and elongation at break (E_B_) were calculated as the average of five strips (length, 20 mm; width, 6 mm; thickness, 45 µm). The transmittance of the films was detected by an ultraviolet–visible (UV–vis) spectrometer on a Shimadzu UV–vis 2501 (Shimadzu, Kyoto, Japan) at 25 °C.

The geometry optimizations for the ground state (S0) of the polymers were performed using the density functional theory (DFT) with the B3LYP hybrid functional and 6-31G (d, p) basis set on Gaussian 09 (version D.01) [22].

### 2.3. Synthesis of the Monomers

#### 2.3.1. Synthesis of HBPA-Based Dinitro Isomers

HBPA (5.0570 g, 0.0200 mol) and 25 mL of DMAc were transferred into a 100 mL ice bathed three-necked flask equipped with a magnetic stirrer, a dropping funnel, a nitrogen inlet and a reflux condenser. Afterward, 60 wt % NaH (1.6400 g, 0.0420 mol) dispersed in 10 mL of DMAc was added dropwise (*caution: the released hydrogen was carefully collected in a hydrogen bag*). After stirring at 0 °C for 45 min, the mixture turned into a gray heterogeneous phase mixture. Subsequently, 4-fluoronitrobenzene (6.0438 g, 0.0420 mol) was added dropwise in 30 min at 0 °C. The reaction is continuously stirred at room temperature for approximately 12 h until no reactant can be detected by TLC. The mixture was slowly poured into rapidly stirred deionized water to precipitate. The residue was recrystallized twice in methanol to obtain a shallow yellow mixed isomers powder (6.94 g, yield, 72%).

#### 2.3.2. Separation of HBPA-Based Dinitro Isomers

**Scheme 1:**
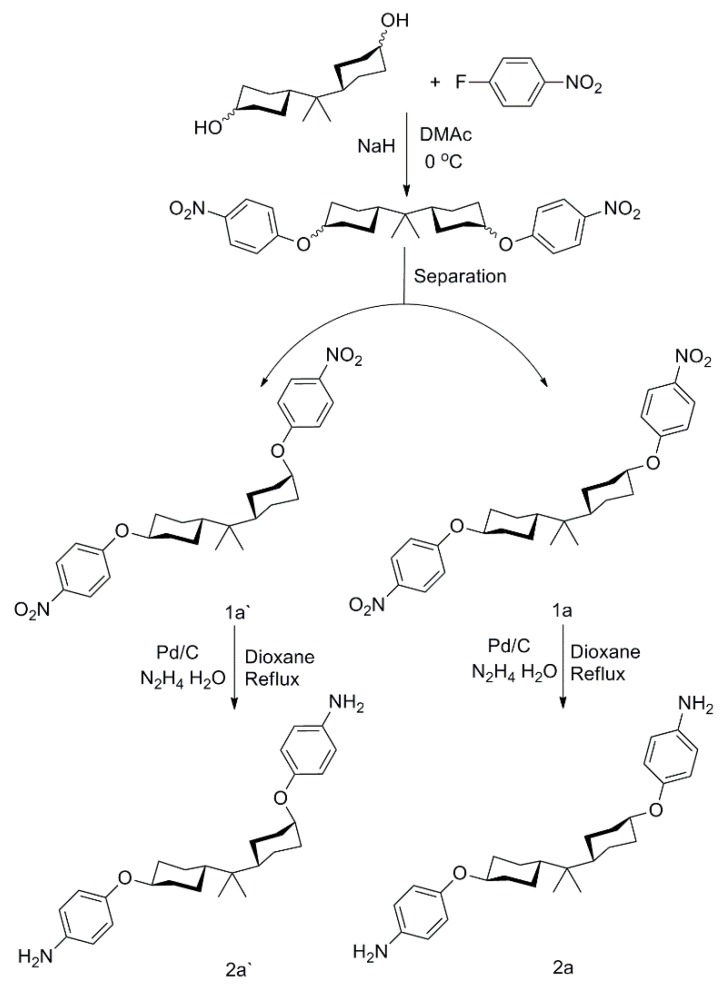
The preparative route of the dinitro (1a′, 1a) and diamine (2a′, 2a) isomers.

**Scheme 2:**
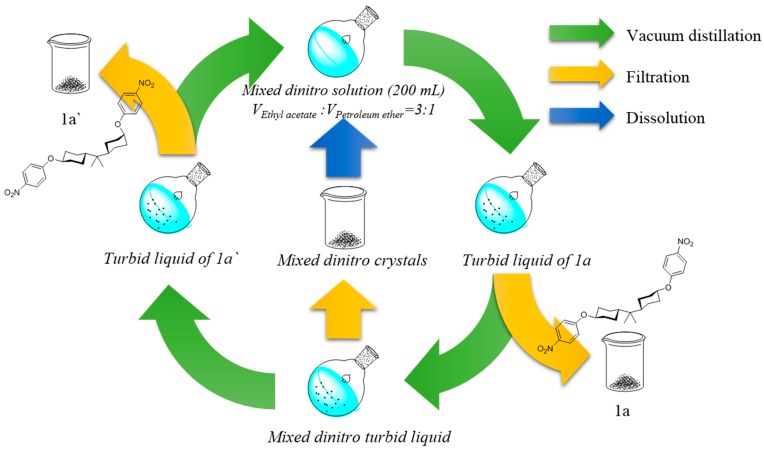
The separation route of dinitro (1a′, 1a).

HBPA as shown in Scheme 2, HBPA based dinitro isomers (5.00 g), ethyl acetate (150 mL) and petroleum ether (30 mL) were transferred into a 500 mL round bottom single-necked flask. After gently shaking the flask, the solid dissolved and the solvent was slowly distilled off using a rotary evaporator. As the amount of solvent concentrated, slender crystals gradually precipitated, the precipitated crystals were filtered once per 30 mL of distilled solvent. As a result, there were five parts of crystals, namely *A*, *B*, *C*, *D*, *E*, corresponding to the volume of the distillation at 30, 60, 90, 120, 150 mL, respectively. ^1^H NMR spectra and DSC traces of these crystals illustrated that *A* was (*trans, trans*)-bis(4-nitrophenoxy)HBPA, corresponding to 1a in Scheme 1, and *E* was (*cis, trans*)-bis(4-nitrophenoxy)HBPA, corresponding to 1a′ in Scheme 1. *B*, *C* and *D* were mixtures of the two isomers. For 1a′, M. p. 148-150 °C. Yield, 55%. ^1^H NMR (300 MHz, DMSO-*d*_6_) δ 8.31–8.01 (m, 4H, H^1^), 7.14 (d, *J* = 9.3 Hz, 4H, H^2^), 4.80 (s, 1H, H^3^), 4.44 (t, *J* = 10.5 Hz, 1H, H^3′^), 2.13 (d, *J* = 10.8 Hz, 2H, H^4^), 2.01 (d, *J* = 13.1 Hz, 2H, H^4′^), 1.74 (d, *J* = 11.5 Hz, 2H, H^5^), 1.54 (dd, *J* = 31.7, 11.4 Hz, 4H, H^6^), 1.25 (ddd, *J* = 21.2, 17.0, 8.3 Hz, 8H, H^7+8^), 0.81–0.65 (m, 6H, H^9^). ^13^C NMR (75 MHz, DMSO-*d*_6_) δ 162.94 (d, *J* = 14.9 Hz, C^1^), 140.35 (s, C^2^), 125.93 (s, C^3^), 115.97 (s, C^4^), 115.60 (s, C^4^), 76.76 (s, C^5^), 72.13 (s, C^5′^), 43.10–42.75 (m, C^6^), 42.39 (d, *J* = 35.9 Hz, C^6^), 36.51 (s, C^7^), 31.72 (s, C^8^), 29.46 (s, C^9^), 24.20 (s, C^10^), 20.44 (s, C^11^).

#### 2.3.3. Synthesis of (*cis, trans*)-bis(4-aminophenoxy)HBPA (2a′)

The compound 1a′ (4.8257 g, 0.0100 mol), 10% Pd/C (0.2500 g) and dioxane (25 mL) were transferred into a 100 mL, round bottle, three-neck flask outfitted with a condenser, a dropping funnel, a nitrogen inlet and a magnetic stirrer. The mixture was heated to reflux under an inert atmosphere. Subsequently, 80% hydrated hydrazine (6.2500 g, 0.1000 mol) was added dropwise for approximately 30 min. The reaction mixture continued to reflux for an additional 2 h until no reactant can be detected by TLC. The reaction mixture was filtered without cooling, and the mother liquor was directly poured into deionized water to precipitate off-white crystals. Yield: 98%. M. p. 178–182 °C. ^1^H NMR (300 MHz, DMSO-*d*_6_) *δ* 6.63 (tt, *J* = 5.5, 2.7 Hz, 4H, H^1^), 6.56–6.33 (m, 4H, H^2^), 4.60 (s, 4H, H^3^), 4.27 (s, 1H, H^4′^), 3.88 (t, *J* = 10.4 Hz, 1H, H^4^), 2.05 (d, *J* = 9.9 Hz, 2H, H^5^), 1.91 (s, 2H, H^5′^), 1.70 (d, *J* = 11.2 Hz, 2H, H^6^), 1.53–0.89 (m, 12H, H^7+8+9^), 0.80–0.64 (m, 6H, H^10^). ^13^C NMR (75 MHz, DMSO-*d*_6_)) *δ* 148.38 (s, C^1^), 148.10 (s, C^1^), 142.53 (d, *J* = 6.6 Hz, C^2^), 118.03 (s, C^3^), 117.48 (s, C^3^), 114.85 (d, *J* = 1.5 Hz, C^4^), 76.98 (s, C^5^), 71.95 (s, C^5′^), 42.98 (s, C^6^), 42.53 (s, C^6^), 36.45 (s, C^7^), 32.50 (s, C^8^), 29.81 (s, C^9^), 24.40 (s, C^10^), 20.45 (s, C^11^). HRLC-MS, m/z: [M+H]^+^, calculated for C_27_H_39_N_2_O_2_, 423.3012, found, 423.3018.

### 2.4. Synthesis of Polyimide Films

**Scheme 3:**
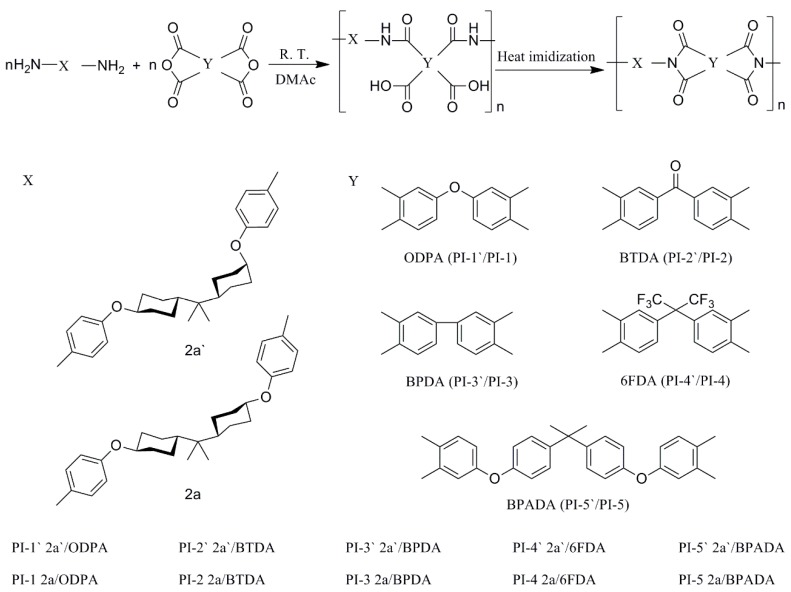
The preparative route of polyimides.

As displayed in Scheme 3, all polyimide films were prepared via a conventional two-step method as previously reported [21]. As a representative for PI-1, 2a (0.8458 g, 0.0020 mol) and ODPA (0.6200 g, 0.0020 mol) were transferred to a 50 mL three neck flask equipped with a mechanical stir under nitrogen protection, a calculated amount of DMAc was added to make the solid content at 15%. The resulting polyamic acid (PAA) solution was continuously stirred at room temperature for 24 h, followed by the heat imidization of viscous PAA solution cast onto a flat glass plate under a gradient heating temperature (80 °C for 1 h, 150 °C for 1 h, 180 °C for 1 h, 250 °C for 1 h, 280 °C for 1 h) in the vacuum tube furnace. No control over the cooling process was carried out after the heating process. After immersing in deionized water, a free-standing film could be peeled off from the glass plate. Similarly, PI-1′ (2a′/ODPA), PI-2 (2a/BTDA), PI-2′ (2a′/BTDA), PI-3 (2a/BPDA), PI-3′ (2a′/BPDA), PI-4 (2a/6FDA), PI-4′ (2a′/6FDA), PI-5 (2a/BPADA), PI-5′ (2a′/BPADA) were obtained by the method mentioned above.

## 3. Results and discussion

### 3.1. Monomers Synthesis and Characterization

**Figure 1:**
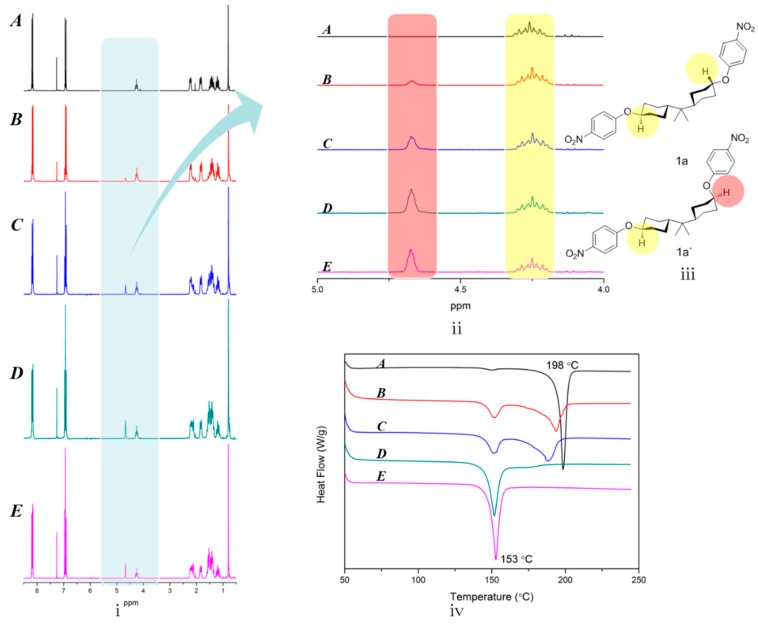
(**i**) The ^1^H NMR spectra precipitated crystals *A*, *B*, *C*, *D*, *E,* corresponding to the volume of the distillation at 30 mL (***A***), 60 mL (***B***), 90 mL (***C***), 120 mL (***D***), 150 mL (***E***); (**ii**) Magnification of the chemical shift at 4–5 ppm in part i; (**iii**) Chemical structure of 1a′ and 1a; (**iv**) DSC curves of crystal *A*, *B*, *C*, *D*, *E*.

As shown in Scheme 1, mixed dinitro monomers were gently synthesized via nucleophilic substitution reaction between HBPA and 4-fluoronitrobenzene. The separation route of the crude product was concisely illustrated in Scheme 2. According to their solubility difference in specifically mixed solvents (V_ethyl acetate_:V_petroleum ether_ = 3:1), as the amount of solvent concentrated, crystal with relatively poor solubility was preferentially precipitated. As a result, five parts of crystals were separately filtered and characterized by ^1^H NMR and DSC. As described in Figure 1, for crystal *A*, resonances at 4.26 ppm appeared the protons of *axial*, *axial* C–H (marked by the yellow circle), demonstrating that crystal *A* was dinitro 1a. Similarly, in the case of crystal *E*, the proton of *equatorial* C–H (marked by the red circle) exhibited resonances at 4.80 ppm and the proton of *axial* C–H (marked by the yellow circle) performed resonances at 4.44 ppm, and the area ratio of the two protons was 1:1, illustrating that crystal *E* was dinitro 1a′. Whereas for crystal *B, C* or *D,* every ^1^H NMR spectrum exhibited two kinds of proton resonances (*axial* C–H near 4.44 ppm and *equatorial* C–H at 4.80 ppm) in unequal content, implying that each crystal was a mixture of 1a′ and 1a in different molar ratios. Further, DSC curves indicated that dinitro 1a and dinitro 1a′ owned melting points at 198 °C (*A*) and 153 °C (*E*), respectively. Whereas the others exhibited two melting points, illustrating that *B*, *C*, *D* were mixtures, and this was consistent with the ^1^H NMR results. It is confusing that HBPA owned three isomers, but we only obtained two isomers (1a′ and 1a), which might be attributed to the fact that the (*cis, cis*)-HBPA based dinitro isomer was removed in the recrystallization process. Both 2a′ and 2a were readily achievable after the reduction of 1a′ and 1a by hydrazine hydrate and Pd/C.

**Figure 2:**
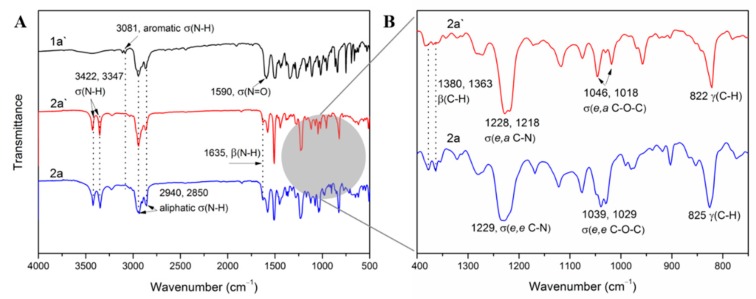
(**A**) The FTIR spectra of 1a′, 2a′ and 2a; (**B**) The infrared fingerprint area spectra (1400–750 cm^−1^) of 2a and 2a′. (*Note: σ: Stretching vibration; β: In-plane bending vibration; γ: Out-of-plane bending vibration.*).

**Figure 3:**
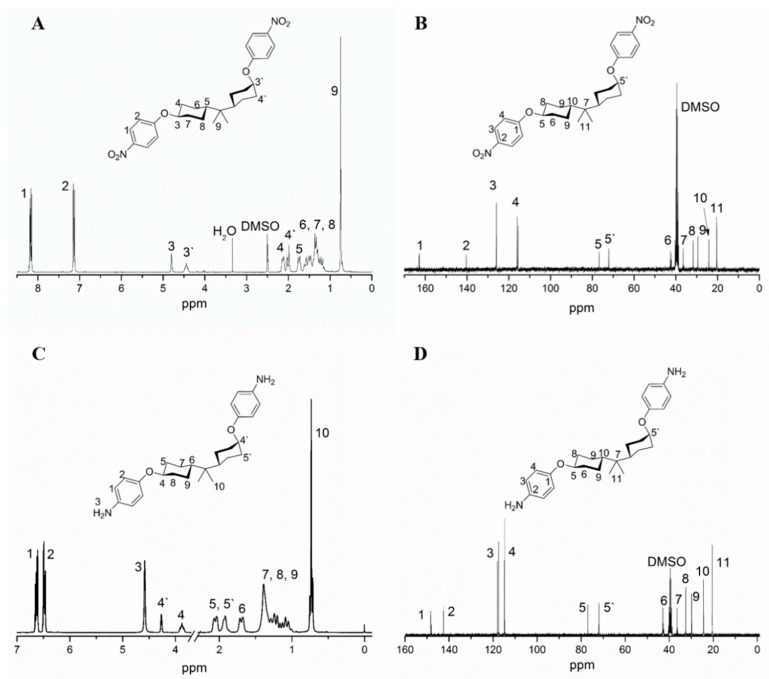
(**A**) The ^1^H NMR spectrum of 1a′; (**B**) ^13^C NMR spectrum of 1a′; (**C**) ^1^H NMR spectrum of 2a′; (**D**) ^13^C NMR spectrum of 2a′.

**Figure 4:**
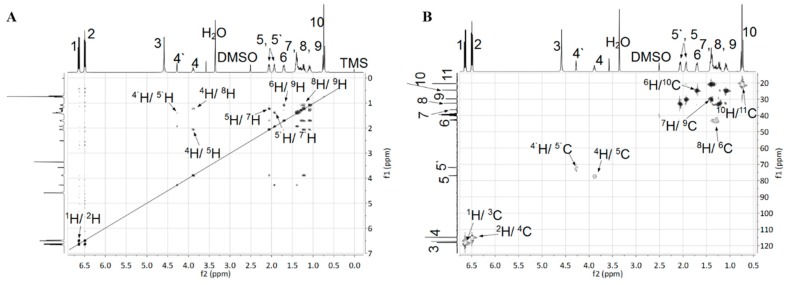
(**A**) The 500 MHz correlation spectrum (COSY) of 2a′; (**B**) 500 MHz heteronuclear single quantum correlation (HSQC) spectrum of 2a′.

The chemical structure of monomers was characterized by HRLC-MS, FTIR, ^1^H NMR, ^13^C NMR and 2D NMR. As depicted in Figure 2, characteristic absorption peaks near 3081 cm^−1^ (aromatic σ(C–H)), 822, 825 cm^−1^ (aromatic γ(C–H)) proved the presence of a para-phenyl structure, and peaks near 2940 cm^−1^, 2850 cm^−1^ (asymmetrical and symmetrical aliphatic σ(C–H)), 1380, 1363 cm^−1^ (β(C–H) of –C(CH_3_)_2_–) indicated that an HBPA moiety was successfully embedded in the diamine. Moreover, the appearance of characteristic absorption peaks around 3422, 3347 cm^−1^ (asymmetrical and symmetrical σ(N–H)), 1635 cm^−1^ (β(N–H)) and the disappearance of peaks around 1590 cm^−1^ (asymmetrical σ(N=O)) illustrated the thorough reduction of 1a′. Additionally, infrared fingerprint area spectra (1400~750 cm^−1^) of 2a and 2a′ were utilized to distinguish their conformation difference. As indicated in Figure 2B, *equatorial*, *equatorial* C–O in 2a exhibited shoulder σ vibrations at 1039, 1029 cm^−1^, while this shoulder split into two independent spikes near 1046 cm^−1^ (*axial* C–O) and 1018 cm^−1^ (*equatorial* C–O) in 2a′. The significant absorption difference between *axial* C–O and *equatorial* C–O was probably attributed to their different steric hindrance: the *axial* C–O may suffer a larger steric hindrance from *axial* C–H in C3 and C5 of HBPA moiety (Figure S1), resulting in a higher absorption wavenumber. A similar situation can be found between σ (C–N) of 2a and 2a′. Further, compared with ^1^H NMR spectrum of 1a′ in Figure 3A, the appearance of the proton resonance at 4.60 ppm (–NH_2_) in Figure 3C also demonstrated the reduction of 1a′, which was consistent with the FTIR results. Moreover, compared with ^1^H and ^13^C NMR of 2a in Reference [21], the appearance of the proton 4′ at 4.27 ppm in Figure 3C and carbon atom 5′ at 71.95 ppm in Figure 3D provided solid evidence for the existence of *axial* C–O in 2a′. Meanwhile, the ^1^H and ^13^C NMR spectra of 1a′ shown in Figure 2A and 2B also presented unambiguously assignment of each proton and carbon atom. To fully accomplish the proton and carbon peak assignment of 2a′, 500 MHz ^1^H-^1^H COSY and ^1^H-^13^C HSQC spectra shown in Figure 4A and B were respectively carried out. As displayed in Figure 4A, the cross-peaks H^1^/H^2^, H^4′^/H^5′^, H^4^/H^5^, H^4^/H^8^, H^5^/H^7^, H^5′^/H^7′^, H^6^/H^9^ and H^8^/H^9^ found in the COSY spectrum at 6.63/6.56, 4.27/1.91, 3.88/2.05, 3.88/1.24, 2.05/1.39, 1.91/1.34, 1.70/1.09 and 1.24/1.09 ppm illustrated the existence of H^1^, H^2^, H^4^, H^4′^, H^5^, H^5′^, H^6^, H^7^, H^8^ and H^9^, respectively. In addition, corresponding carbon atoms linked to hydrogen atoms were also found by the HSQC spectrum. As exhibited in Figure 4B, the HSQC signals at 6.63/118.03, 6.56/114.85, 4.27/71.95 and 3.88/76.98 ppm were ascribed to H^1^/C^3^, H^2^/C^4^, H^4′^/C^5′^ and H^4^/C^5^, while H^6^/C^10^, H^7^/C^9^, H^8^/C^6^ and H^10^/C^11^ signals were found at 1.70/24.40, 1.39/29.81, 1.24/42.53 and 0.73/20.45 ppm, respectively.

### 3.2. Polyimides Synthesis

**Figure 5:**
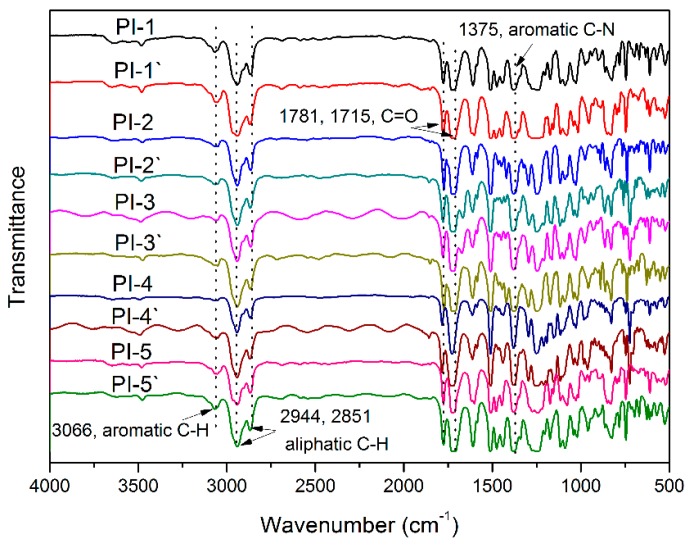
The FTIR spectra of polyimides.

**Figure 6:**
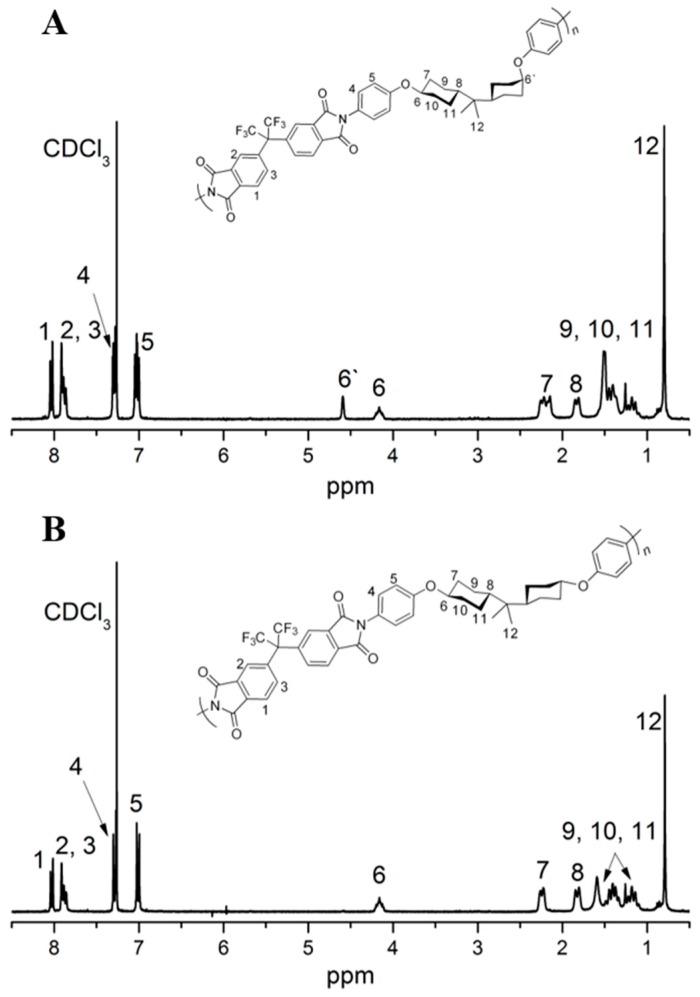
The ^1^H NMR spectra of PI-4′ (2a′/6FDA) (**A**) and PI-4 (2a/6FDA) (**B**).

As described in Scheme 3, all the polyimides were obtained via soluble PAA precursors, followed by heat imidization under a gradient temperature. Characterizations including FTIR and ^1^H NMR spectra were employed to confirm the chemical structure of polyimides. As listed in Table 1, the viscosity of PAA solution exhibited a range of 0.87–1.53 dL/g at 25 °C, and M_n_ and polydispersities (M_w_/M_n_) of the corresponding polyimides covered a range of 5.2 × 10^4^–9.1 × 10^4^ and 1.33–1.76, respectively, illustrating the relatively high molecular weights. In addition, FTIR spectra of the polyimides are displayed in Figure 5, characteristic absorption peaks near 2944 and 2851 cm^−1^ (asymmetric and symmetric σ(C–H) in –CH_2_) demonstrated the existence of HBPA segments. The appearance of peaks near 1781 cm^−1^ (asymmetrical σ(C=O)), 1715 cm^−1^ (symmetrical σ(C=O)) and 1379 cm^−1^ (σ(C–N)) and the disappearance of peaks around 3220−3450 cm^−1^ (σ(N–H)) and 1580−1620 cm^−1^ (β(N–H)) elucidated the thorough imidization of PAA. Besides, ^1^H NMR spectra of PI-4′ and PI-4 were also carried out as representatives, as shown in Figure 6; characteristic resonances near 0.70–0.94 ppm demonstrated the presence of isopropyl, while resonances around 2.10–2.18 ppm, 1.93–1.75 ppm and 1.68–1.04 ppm illuminated the existence of -CH_2_- in the HBPA moiety. Notably, ^1^H NMR spectrum of PI-4′ (Figure 6A) containing (*cis, trans*)-HBPA fragment presented an equal integral area of proton 6′ resonance (*equatorial* C–H bond at 4.59 ppm) and proton 6 resonance (*axial* C–H bond at 4.16 ppm), and ^1^H NMR spectrum of PI-4 (Figure 6B) containing a (*trans, trans*)-HBPA segment exhibited the only proton 6 resonances (*axial, axial* C–H bond at 4.16 ppm). This suggested that no conformational changes took place during the preparation of HBPA based polyimides.

### 3.3. Thermal Properties

**Figure 7:**
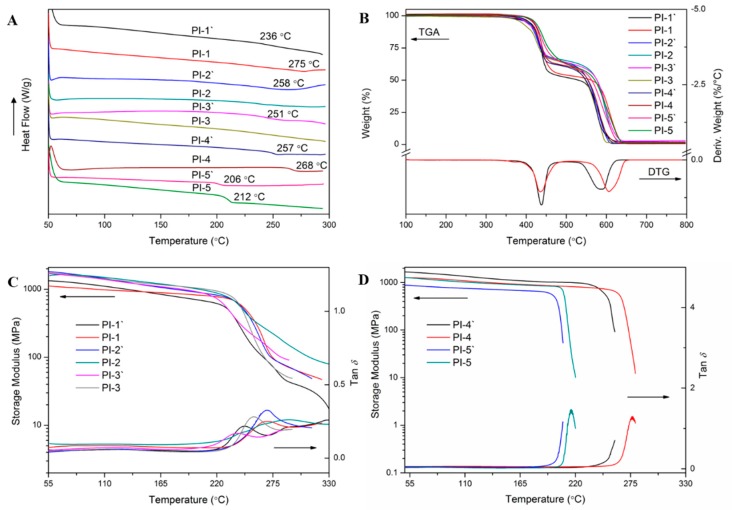
(**A**) The DSC traces of the second heating cycle of polyimides, arrow direction represents heat release direction; (**B**) TGA and DTG curves of polyimides in the air; (**C**) and (**D**) Storage modulus and tan *δ* curves of polyimide films measured by DMA.

**Figure 8:**
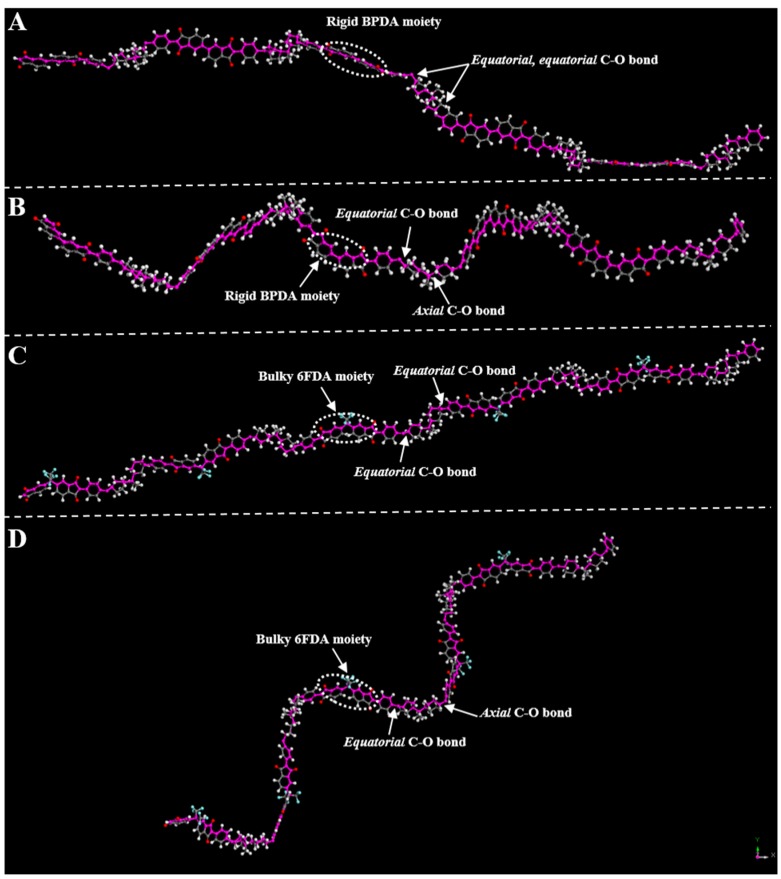
The geometry optimizations by Gaussian 09 for the ground state (S_0_) of representative polyimides with five repeating units. (**A**) PI-3; (**B**) PI-3′; (**C**) PI-4; (**D**) PI-4′.

To fully evaluate the thermal properties of polyimides, measurements including DSC, DMA and TGA were carried out and the results are summarized in Table 2. As shown in Figure 7, the T_g_ of these polyimides was 206–275 °C by DSC (Figure 7A) and 208–292 °C by DMA (Figure 7C,D), respectively. Generally, according to the Kuhn and Paul J. Flory theories [23], the T_g_ of polymers are mainly determined by the rigidity of repeating units and the molecular chain packing. No apparent T_g_ of PI-2 or PI-3 was found in DSC curves, implying that the HBPA moiety possessed a certain degree of rigidity [24]. As shown in the DMA curves, both PI-1 (2a/ODPA, 247 °C) and PI-3 (2a/BPDA, 256 °C) possessed a lower T_g_ than that of PI-4 (2a/6FDA, 276 °C), which can be attributed to the larger steric limitations imparted by the sp^3^-swivel moiety of trifluoromethyl in 6FDA [25]. The lowest T_g_ of PI-5 (2a/BPADA, 217 °C) was probably due to the chain flexibility promoted by the rotational freedom of the ether linkages in the BPADA unit. Curiously, in our previous study, it was concluded that hexafluoroisopropyl in 6FDA possessed a larger steric hindrance than carbonyl in BTDA [26], nevertheless, the T_g_ of PI-4 exhibited a temperature that was 16 °C lower than that of PI-2 (2a/BTDA, 292 °C). This may be attributed to the crosslinking of benzophenone C=O groups during thermal imidization process, resulting in the higher T_g_ [27]. Notably, for a given dianhydride, the T_g_ of polyimides containing the (*trans, trans*)-HBPA moiety is always higher than that of polyimides containing the (*cis, trans*)-HBPA moiety, e.g., PI-1 > PI-1′ (2a′/ODPA, 247 °C), PI-2 > PI-2′ (2a′/BTDA, 270 °C). This was possibly ascribed to the *axial, equatorial* C-O in (*cis, trans*)-HBPA unit possessing an asymmetrical structure, e.g., the monomer polymerization form may be head to tail, head to head or tail to tail [28], this can lead to looser molecular chain packing than that of *equatorial, equatorial* C–O in (*trans, trans*)-HBPA segment, resulting in the lower T_g_ of polyimides. This can be evidenced by the geometric optimization (Gaussian 09) results of the representative polyimides, as shown in Figure 8, the molecular chains of representative polyimides (PI-3′ and PI-4′) containing the *axial, equatorial* C–O were more distorted and flexible than that of polyimides (PI-3 and PI-4) containing *equatorial, equatorial* C–O.

**Figure 9:**
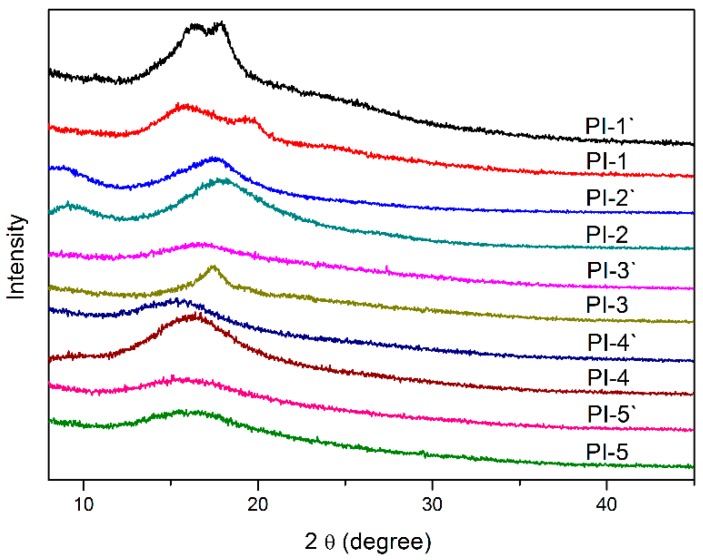
The X-ray film diffraction patterns of polyimides at 25 °C.

Additionally, TGA curves both in N_2_ and air atmospheres are presented in Figure S3 and Figure 7B, respectively, and the results are listed in Table 2. No apparent thermal decomposition could be found before 407 °C in N_2_, 5% weight loss temperature (T_5%_) in N_2_ and air located in the range of 419–434 °C and 387–422 °C, respectively, which indicated their high thermal stability. DTG curves of representative PI-1 presented two peaks, suggesting that the decomposition process of polyimides in the atmosphere went through two stages [29]. The first stage at 378–483 °C corresponded to the thermal decomposition of an aliphatic fragment, which was mainly caused by the oxidation of the α-position carbon in HBPA residues [30]. The second stage at a higher temperature (531–654 °C) corresponded to the decomposition of aromatic moiety [31]. Interestingly, in the second decomposition stage, polyimides containing (*trans, trans*)-HBPA units always owned a higher decomposition temperature than that of polyimides containing (*cis, trans*)-HBPA units, for instance, the second decomposition temperature of PI-1 was 561–648 °C, higher than that of PI-1′(531–624 °C). This was probably attributed to the rigid linear polymer chains endowed by *equatorial, equatorial* C–O were relatively stable at higher temperatures, while the distorted and flexible *axial, equatorial* C–O loosened the molecular chains packing, making the polymer susceptible to be oxidized. This can be evidenced by WAXD results, as shown in Figure 9, for a given dianhydride, polyimides containing the (*cis, trans*)-HBPA moiety always had a larger molecular chain d-spacing than that of polyimides containing (*trans, trans*)-HBPA moiety, and this was in accordance with the geometry optimization results (Figure 8) of the polymer chains.

### 3.4. Mechanical and Morphological Properties

The mechanical properties of polyimides were carried out at 23 ± 2 °C and a relative humidity of 50 ± 5% based on ASTM D882-02, and the results are listed in Table 3. The polyimide films showed the tensile strength (T_S_), tensile modulus (T_M_) and elongation at break (E_B_) in the range of 72–117 MPa, 1.1–3.2 GPa and 8.3–12.6%, respectively. Classically, the embedded aliphatic chains into the backbone of polyimides led to the compromise of their mechanical property [32], however, most of the polyimide films owned comparable mechanical properties with that of the reported aromatic polyimides [33], suggesting that the HBPA moiety owned the parallel stiffness and toughness with the aromatic ones. The highest T_S_ of PI-2′ (117 MPa) may have a relationship with the crosslinking of the benzophenone C=O groups during thermal imidization, as well as the high molecular weight of the polymer. Whereas, the lowest T_S_ of PI-5 at 72 MPa might be attributed to their relatively bent and distorted structure, resulting in the weaker interaction of molecular chains. For a given dianhydride, no significant differences were found in mechanical properties between polyimides containing the (*cis, trans*)-HBPA moiety and polyimides containing the (*trans, trans*)-HBPA moiety, which may be ascribed to their difference in molecular weights [34].

Wide-angle X-ray Diffraction was used to analyze the morphological structure of the polyimide films. As displayed in Figure 9, one halo was observed with 2 θ varying 15.46° to 17.91°, and the calculated d-spacing of molecular chains was 4.95–5.73 Å, implying that all the polyimide films were amorphous, which was consistent with the DSC results [35]. Interestingly, when the dianhydride was fixed, polyimides containing the (*cis, trans*)-HBPA moiety always possessed larger d-spacing than that of polyimides containing the (*trans, trans*)-HBPA moiety, which could be attributed that the *axial, equatorial* C–O in the backbone of polyimides which could lead to looser molecular chain stacking than that of *equatorial, equatorial* C–O.

### 3.5. Solubility

A solubility test was carried out at 25 °C by dissolving 10 mg of polyimide films in 1 mL of solvent for 24 h and the results are summarized in Table 4. As can be seen, most of the polyimides exhibited outstanding solubility not only in common polar solvents such as DMF, DMAc and NMP, but in low boiling solvents like CH_2_Cl_2_ and CHCl_3_, which was rarely seen among other semi-aromatic polyimides [36]. Such a favorable solubility can be ascribed to two aspects: on the one hand, the twisted and flexible ether linkages in ODPA and BPADA units, as well as the bulky hexafluoroisopropyl in 6FDA efficiently weakened the intermolecular cohesive force [7]. On the other hand, the flexuous “chair shaped conformation” of the HBPA moiety in the backbone of polyimides promoted the loose packing of molecular chains [37]. The inferior solubility of PI-2′ and PI-2 might have a close relationship with the crosslinking of benzophenone C=O groups, and the rigid and linear structure of BPDA may lead to the poor solubility of PI-3′ and PI-3. Interestingly, polyimides containing (*cis, trans*)-HBPA moiety had a better solubility than polyimides containing (*trans, trans*)-HBPA moiety, e.g., PI-1′ > PI-1, PI-2′ > PI-2, which can be attributed to *axial, equatorial* C–O which possessed larger space than that of *equatorial, equatorial* C–O. However, no clear distinctions between PI-4′ and PI-4, PI-5′ and PI-5 were found, which might be a result of the “already loosened” molecular chains caused by twisted and bulky segments (–O–, –CF_3_). Notably, easy processability using common organic solvents, especially low boiling solvents, made these polymers suitable for solution processing technology [38].

### 3.6. Optical Properties

**Figure 10:**
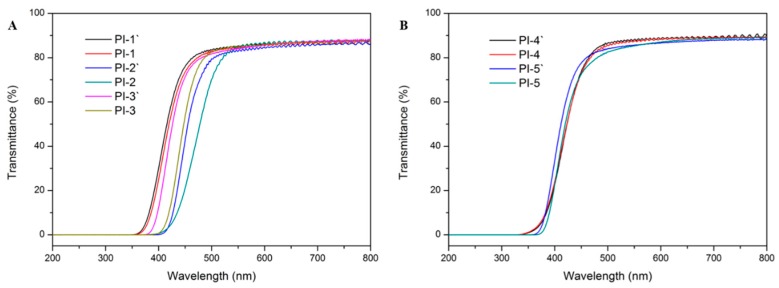
The UV-vis curves of polyimides.

**Figure 11:**
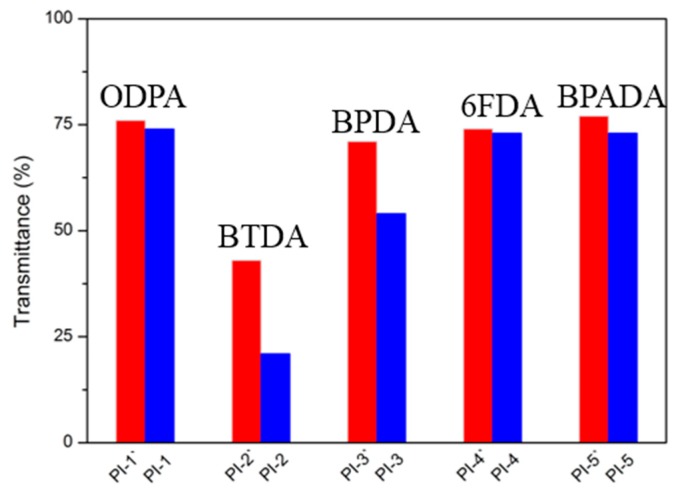
The transmittance of polyimides at 450 nm.

UV-vis spectroscopy (Figure 10) was utilized to characterize the optical transparency of polyimide films with a thickness of approximately 10 μm. As summarized in Table 3, for PI-(1-5), PI-1 (74%), PI-4 (73%) and PI-5 (73%) exhibited a higher transmittance than PI-2 (21%) and PI-3 (54%). This could be attributed to two aspects. Firstly, it should be the integration of the HBPA moiety in the backbone of polyimides: the lower electron-donating ability of the alicyclic fragments weakened the inter- and intramolecular CT interaction [39]. Secondly, the existence of flexible ether linkages and bulky substituent effectively loosened the molecular chain stacking and led to the impediment of the CT interaction. The rigid BPDA residue in PI-3 and the crosslinking of benzophenone C=O groups in PI-2 were positive to the formation of CT interaction, resulting in the coloration of the polyimides. Additionally, Figure 11 describes the transmittances of polyimides at 450 nm, as can be seen, for a given dianhydride, e.g., ODPA, BPADA or 6FDA; no significant difference in transmittance could be found between polyimides containing (*cis, trans*)-HBPA moiety and polyimides containing (*trans, trans*)-HBPA moiety, which could be the reason that the twisted and flexible ether linkages in ODPA and BPADA residues, and bulky –(CF_3_)_2_ in the 6FDA fragment led to “already weakened” CT interactions. Therefore, the impact differences of the *axial, equatorial* C–O and *equatorial, equatorial* C–O are no longer major contributions in obstructing the CT interaction, and it was also a similar case in the solubility of polyimides. However, when it comes to rigid and stiff dianhydrides, e.g., BPDA or BTDA, the influence of *axial, equatorial* C–O and *equatorial, equatorial* C–O on the transmittance of the polyimides can be reflected, which was possibly owing to the *axial, equatorial* C–O possessing advantage in reducing the CT interaction because of their larger molecular chain d-spacing imparted by *axial* C–O.

## 4. Conclusions

In this work, an HBPA based dinitro isomer mixture was synthesized via nucleophilic substitution, and dinitro 1a′ and 1a were successfully separated by vacuum distillation under the monitor of DSC and ^1^H NMR. Corresponding diamines were respectively polycondensed with five commercial dianhydrides via a two-step thermal imidization to obtain PI-(1′-5′) and PI-(1-5). The chemical structure of monomers and polyimides were identified by HRLC-MS, FTIR, ^1^H NMR, ^13^C NMR and 2D NMR. The main conclusions were as follows:

(1) All the polyimides could afford flexible, tough, and transparent films, and most of them were readily soluble not only in common polar solvents, e.g., DMAc, DMF, but in low boiling point solvents such as chloroform and dichloromethane.

(2) ^1^H NMR spectra of the polyimides indicated that isomers of HBPA moieties had no conformation changes during the preparation of polymers.

(3) For a given dianhydride, it was concluded that PI-(1-5) possessed better thermal stability, e.g., higher T_g_ and a thermal decomposition temperature than that of PI-(1′-5′), this can be attributed that the *equatorial* C–O in PI-(1-5) promoted denser and more regular molecular chain stacking, as can be evidenced by the WAXD and geometric optimization results.

(4) When the dianhydride was ODPA, BPADA or 6FDA, no apparent difference was found either in the transmittance or solubility between the two series of polyimides. This could be the reason that the twisted and flexible ether linkages, as well as a bulky substituent, led to “already weakened” inter- and intramolecular CT interactions and cohesive force. However, when it came to rigid and stiff dianhydride, e.g., BPDA, PI-3′ took an obvious advantage over PI-3 on aspects of transmittance and solubility, which was possibly owing to the larger molecular chain d-spacing imparted by *axial* C–O.

Their satisfactory overall performance, particularly their excellent transmittance and processability combined with good mechanical properties made these polymers ideal candidates for optoelectronic device substrates. Notably, the successful separation of HBPA based dinitro isomers, as well as the successful integration of (*trans, trans*)-HBPA and (*cis, trans*)-HBPA moieties into the backbone of polyimides may provide a new horizon to develop functional polyimides.

## Figures and Tables

**Table 1:** The viscosity of the polyamic acid solution and molecular weight of polyimides.

Code	(η)(dL/g) ^a^	Molecular weights ^b^		
	PAA	M_n_ /10^4^	M_w_ /10^4^	M_w_/M_n_
PI-1′	1.23	8.8	15.0	1.70
PI-1	1.23	7.0	9.8	1.40
PI-2′	1.51	− ^c^	−	−
PI-2	1.46	−	−	−
PI-3′	1.39	−	−	−
PI-3	0.93	−	−	−
PI-4′	1.03	8.0	14.1	1.76
PI-4	1.53	9.1	14.0	1.54
PI-5′	0.94	5.6	7.7	1.38
PI-5	0.87	5.2	6.9	1.33

^a^ Inherent viscosities of PAA in DMAc were measured at 25 °C with a concentration of 0.5 g dL^−1^. ^b^ Measured by GPC in DMF with polystyrene as the standard. ^c^ Polyimide film was not soluble in DMF and the molecular weights could not be measured.

**Table 2:** The thermal properties analyzed by DSC, DMA, TGA.

Polyimides	T_g_ (°C)		T_5%_ (°C) ^c^		T_10%_ (°C) ^c^		R_W_ (°C) ^d^
	DSC ^a^	DMA ^b^	In N_2_	In the Air	In N_2_	In the Air	
PI-1′	236	247	424	408	431	422	24
PI-1	275	267	426	407	433	421	23
PI-2′	258	270	419	400	427	416	34
PI-2	− ^e^	292	425	404	432	419	35
PI-3′	251	241	428	387	436	407	34
PI-3	−	256	434	399	440	418	32
PI-4′	257	260	427	408	435	420	20
PI-4	268	276	429	417	437	427	14
PI-5′	206	208	431	419	438	429	26
PI-5	212	217	432	422	440	433	25

^a^ Obtained at the baseline shift in the second heating DSC traces, the temperature was raised at 10 °C/min with a nitrogen flow rate at 50 mL/min. ^b^ Measured by DMA with a heating rate of 5 °C/min and a load frequency of 1 Hz in film tension geometry in air. ^c^ 5% and 10% weight loss temperatures measured by TGA, the temperature was raised at 10 °C/min with a nitrogen flow rate at 50 mL/min. ^d^ Residual weight retention at 800 °C by TGA, the temperature was raised at 10 °C/min with a nitrogen flow rate at 50 mL/min. ^e^ ND, not detected.

**Table 3:** The mechanical, optical properties and X-ray diffraction spacing of polyimide films.

Polyimides	T_S_^a^ (MPa)	T_M_^b^ (GPa)	E_B_^c^ (%)	Transmittance (%) ^e^	λ_cut-off_ (nm) ^f^	2 θ (deg)	X-ray Diffraction (Å) ^g^
PI-1′	105 ± 2.7 ^d^	3.2 ± 0.2	9.2 ± 2.1	76	347	17.08	5.19
PI-1	112 ± 1.0	2.3 ± 0.1	11 ± 1.0	74	349	17.73	4.99
PI-2′	117 ± 4.2	3.1 ± 0.5	8.8 ± 1.4	43	390	17.56	5.05
PI-2	103 ± 3	2.4 ± 0.6	8.6 ± 1.9	21	366	17.91	4.95
PI-3′	108 ± 6.6	2.9 ± 0.9	8.3 ± 1.4	71	370	16.83	5.26
PI-3	83 ± 2.8	2.2 ± 0.3	10.6 ± 2.5	54	380	17.41	5.09
PI-4′	100 ± 6.2	2.5 ± 0.2	7.5 ± 0.7	74	328	15.46	5.73
PI-4	101 ± 4.1	2.5 ± 0.1	7.5 ± 0.3	73	327	16.44	5.39
PI-5′	91 ± 9.9	1.1 ± 0.8	12.6 ± 1.0	77	354	15.81	5.60
PI-5	72 ± 3.9	2.1 ± 0.1	10 ± 1.0	73	362	16.09	5.50

^a^ T_S_, Tensile strength. ^b^ T_M_, Tensile modulus. ^c^ E_B_, Elongation at break. ^d^ 2.7, standard deviation. ^e^ Transmittance at 450 nm, film thickness at 10 μm. ^f^ Cut-off wavelength. ^g^ X-ray diffraction: the average distance between the molecular chains (d-spacing) was calculated by the Bragg equation nλ = 2 d sinθ (λ = 1.5406 Å).

**Table 4:** The solubility properties of the polyimide films.

Solvents	PI-1′	PI-1	PI-2′	PI-2	PI-3′	PI-3	PI-4′	PI-4	PI-5′	PI-5
DMAc ^a^	+−	+−	−	−	−	−	++	+−	++	++
DMF	++	+−	−	−	−	−	++	++	++	+−
NMP	++	++	+−	−	+−	−	++	++	++	++
CH_2_Cl_2_	++	+−	+−	−	+−	+−	++	++	++	++
CHCl_3_	++	+−	+−	−	+−	−	++	++	++	++
THF	−	−	−	−	−	−	+−	−	+−	+−

^a^ DMAc = *N,N*-dimethylacetamide; DMF = *N,N*-dimethylformamide; NMP = *N*-methyl-2-pyrrolidone; THF = Tetrahydrofuran. ++, soluble at 25 °C; +−, partial soluble; −, insoluble.

## Supplementary Materials

The following are available online at https://www.mdpi.com/2073-4360/11/5/854/s1, Figure S1: Conformation structure of HBPA isomers, including (*trans, trans*)-HBPA, (*cis, cis*)-HBPA, (*cis, trans*)-HBPA. Figure S2: (A) GC-MS of HBPA. Component α: (*cis, cis*)-HBPA, 8%; Component β: (*cis, trans*)-HBPA, 45%; Component γ (*trans, trans*)-HBPA, 47%. (B) Fragment ion peak of component γ. Figure S3: TGA curves of PI-(1-5) and PI-(1′-5′) in N_2_.
