# Supplementary material for: Soluble Polyimides Bearing (cis, trans)-Hydrogenated Bisphenol A and (trans, trans)-Hydrogenated Bisphenol A Moieties: Synthesis, Properties and the Conformational Effect
Source: Polymers (Basel). 2019 May 10;11(5):854. doi: 10.3390/polym11050854 (PMC6571896; doi:10.3390/polym11050854)
Supplement: Supplementary file 1 [file polymers-11-00854-s001.pdf]

Soluble polyimides bearing (*cis, trans*)-hydrogenated bisphenol A and (*trans, trans*)-hydrogenated bisphenol A moieties: synthesis, properties and the conformational effect

Zhiming Mi [a], Shuai Wang [a], Ziwen Hou [a], Zhixiao Liu [a], Sizhuo Jin [a], Xiaowen Wang [a], Daming Wang [a], Xiaogang Zhao [a], Yumin Zhang [b], Hongwei Zhou *[a], Chunhai Chen [a]

[a] *Key Laboratory of High Performance Plastics (Jilin University), Ministry of Education. National & Local Joint Engineering Laboratory for Synthesis Technology of High Performance Polymer. College of Chemistry, Jilin University, Changchun, 130012, P. R. China.*

[b] *College of Chemistry, Jilin University, Changchun, 130012, P. R. China.*

Correspondence to: Hongwei Zhou (E-mail: zhw@jlu.edu.cn).

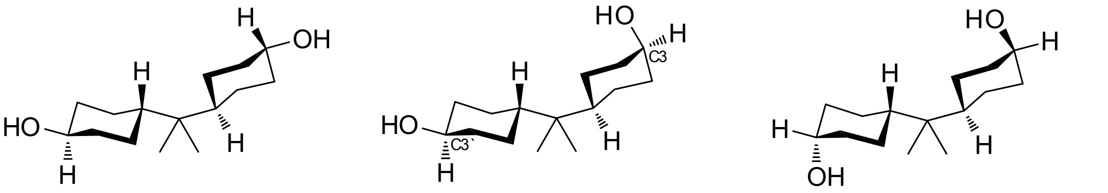

equatorial, equatorial C-O	*equatorial, axial* C-O	*axial, axial* C-O
(*trans, trans*)-HBPA	(*cis, trans*)-HBPA	(*cis, cis*)-HBPA

Figure S1 Conformation structure of HBPA isomers, including (*trans, trans*)-HBPA, (*cis, cis*)-HBPA, (*cis, trans*)-HBPA.

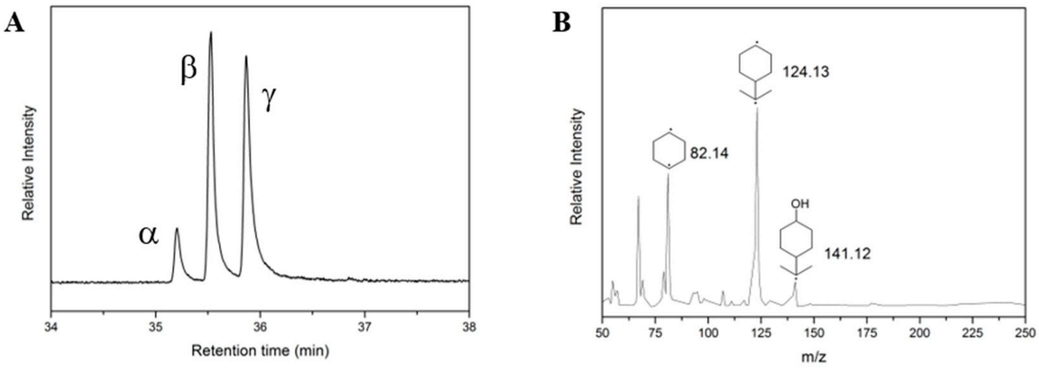

Figure S2 (A) GC-MS of HBPA. Component α: (*cis, cis*)-HBPA, 8%; Component β: (*cis, trans*)-HBPA, 45%; Component γ (*trans, trans*)-HBPA, 47%. (B) Fragment ion peak of component γ.

Condition parameters of GC-MS: Instrument model: GCMS-QP2010, SHIMADZU; Column model: DB-5MASS; Stationary phase: polymethylsiloxane; Mobile phase: Helium. Heating condition:5 °C/min, 50 °C-280 °C.

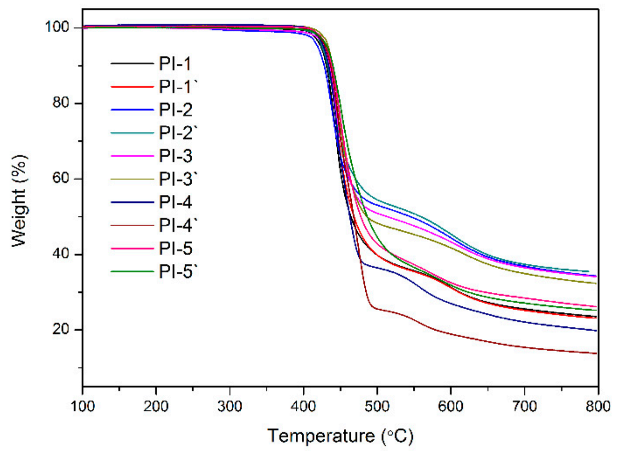

Figure S3 TGA curves of PI-(1-5) and PI-(1`-5`) in N_2.
